# Optimization of Briquette Fuels by Co-Torrefaction of Residual Biomass and Plastic Waste Using Response Surface Methodology

**DOI:** 10.3390/molecules28062568

**Published:** 2023-03-11

**Authors:** Shuai Guo, Lidong Liu, Deng Zhao, Chenchen Zhao, Xingcan Li, Guangyu Li

**Affiliations:** 1 School of Energy and Power Engineering, Northeast Electric Power University, Jilin 132012, China; 2 Harbin Boiler Company Limited, Harbin 150046, China; 3 College of Vehicles and Energy, Yanshan University, Qinhuangdao 066004, China; 4 State Key Laboratory of High-Efficiency Utilization of Coal and Green Chemical Engineering, Ningxia University, Yinchuan 750021, China

**Keywords:** torrefaction, response surface methodology, biomass waste, polypropylene plastic waste, circular economy

## Abstract

Combining biomass, a clean and renewable energy source, with waste plastic, which serves as a good auxiliary fuel, can produce high-quality clean fuel. The performance of biomass-derived fuel can be improved by torrefaction. This study optimized the co-torrefaction of fungus bran and polypropylene (PP) waste plastic to obtain clean solid biofuel with high calorific value and low ash content (AC) using response surface methodology. Two sets of mixed biochars were investigated using a multiobjective optimization method: mass yield–higher heating value–ash content (MY-HHV-AC) and energy yield–ash content (EY-AC). PP increased the heat value, decreased AC, and acted as a binder. The optimal operating conditions regarding reaction temperature, reaction time, and PP blending ratio were 230.68 °C, 30 min, and 20%, respectively, for the MY-HHV-AC set and 220 °C, 30 min, 20%, respectively, for the EY-AC set. The MY-HHV-AC set had properties close to those of peat and lignite. Furthermore, compared with that of the pure biochar, the AC of the two sets decreased by 15.71% and 14.88%, respectively, indicating that the prepared mixed biochars served as ideal biofuels. Finally, a circular economy framework for biobriquette fuel was proposed and prospects for preparing pellets provided.

## 1. Introduction

Because of rapid economic and social development, increasing energy demands, and environmental pollution, the severity of the greenhouse effect, and consequently global warming, is increasing [1]. Biomass energy is an alternative to fossil energy as it is a renewable, clean, and carbon-neutral resource [2]. China is primarily an agricultural country rich in edible fungi resources, producing over 13 million tons of edible fungus waste annually [3]. Fungus bran (FB), which is the waste residue after planting edible fungus, has lignocellulosic biomass. It comprises wood chips (carbon source), wheat bran, and soybean residue (nitrogen source), which are rich in hemicellulose, cellulose, and lignin [4]. Unlike other lignocellulosic biomasses, the growth of cultivated fungi is not affected by seasonal changes; therefore, FB has great potential as a biomass energy source. Except where wood ear mushroom bran is discarded in fields, most of the wood ear mushroom bran is directly burned, which produces heavy smoke, thus resulting in not only resource wastage but also serious environmental damage. Furthermore, plastic usage is widespread in domestic and industrial applications, and its annual global production exceeds 380 million tons [5]. Globally, <20% of waste plastic is recycled. Mostly, it is disposed of in landfills and <10% is combusted for the generation of electricity as an alternative to coal and biomass. However, plastic waste can be an efficient and environmentally friendly auxiliary fuel [5]. Polypropylene (PP), a high-carbon content polymer, is used extensively in disposable products. Furthermore, PP is chlorine-free and has a high calorific value and low ash content (AC) compared to coal and biomass [6]. With the gradual implementation of domestic waste segregation in China since 2020, collecting and supplying a large amount of “white garbage” plastic waste to the energy production system has become possible.

Compared with fossil fuels, biomass feedstock is disadvantageous in terms of its high water content, poor moisture absorption, poor grindability, and low calorific value, which reduce energy utilization and increase inconvenience in its transportation and storage [7]. To overcome these problems, biomass should be pretreated to improve the efficiency of energy conversion. Moreover, compared with other thermal pretreatment methods, such as pyrolysis and hydrothermal carbonization, torrefaction is relatively more stable and energy efficient. Torrefaction is a mild pyrolysis process in which biomass is thermally degraded at 200–300 °C [8]. Post-torrefaction, the H/C and O/C ratios and hemicellulose contents of biomass decrease, whereas cellulose, lignin, and ash contents increase [9]; furthermore, the calorific value, hydrophobicity, and grinding ability of biomass improve, with the performance being closer to that of coal combustion [10].

Many scholars have studied the co-torrefaction of biomass. For example, Kizuka [11] studied the fuel performance of rice straw pellets after the torrefaction of a mixture of rice straw and wood chips and found that when the reaction temperature was 250 °C and 50% wood chips were added, the calorific value of the mixed biochar increased from 16.09 MJ/kg to 16.8 MJ/kg and the energy density increased from 11.53 GJ/m³ to 12.04 GJ/m³ compared to that by the torrefaction of only rice straw; thus, the fuel utilization efficiency increased by using both wood chips and rice straw. Furthermore, Rodríguez [12] found that adding PP during vegetable waste torrefaction increased the calorific value and reduced the AC. Moreover, at a reaction temperature of 250 °C and a reaction time of 15 min, the calorific value of the mixed biochar was 19.14 MJ/kg, the AC was 30.03% when 1% PP was added, and the calorific value increased to 19.66 MJ/kg and the AC decreased to 28.89% when 5% PP was added. Emadi [13] studied the torrefaction of low-density polyethylene (LDPE) and wheat straw and found that 1–10% LDPE increased the calorific value and reduced the AC of the mixture. Obiora [14] reported similar results while studying the torrefaction of wheat straw and barley straw with high-density polyethylene (HDPE). As the proportion of HDPE increased, the calorific value of the mixture increased, and the maximum calorific values of barley and wheat mixtures were 28.34 MJ/kg and 29.78 MJ/kg, respectively; moreover, the ACs in the wheat and barley mixtures decreased from 10.34% to 4.59% and 10.66% to 3.88%, respectively. Chen [15] used discarded chopsticks and discarded plastic spoons as binders for co-torrefaction with coffee grounds and found that the mixed chopsticks and mixed spoons increased the fixed carbon content and calorific value of biofuel, respectively, reaching a maximum of 26.21 MJ/kg with 50% coffee grounds, 30% spoons, and 20% chopsticks; additionally, the mixed biochar had a uniform and continuous surface.

Co-torrefaction is influenced differently by different factors, such as reaction temperature, reaction time, and blending ratio. To optimize the experimental conditions to achieve optimum results, an optimization algorithm should be used to evaluate the torrefaction process. Moreover, the effect of the interaction of operating parameters on the physicochemical properties of biomass should be studied [16]. Consequently, researchers are using the response surface optimization algorithm to optimize the biomass torrefaction process.

Guo [17] optimized the torrefaction of the corn stover using response surface methodology (RSM) and reported that temperature was the most important factor affecting the torrefaction; particularly, the mass yield and calorific value were maximized at a reaction temperature of 242.26 °C, a residence time of 60 min, and a heating rate of 6.28 °C/min. Singh [18] used RSM to optimize the torrefaction of the pigeon pea stalk, under a reaction temperature of 248.2 °C, a reaction time of 60 min, and a heating rate of 16.03 °C/min; consequently, the maximum higher heating value (HHV) and energy yield were 21.15 MJ/Kg and 78.8%, respectively, HHV and fixed carbon content increased by 25.07% and 185.86%, respectively, and oxygen content decreased by 13.05%. Furthermore, to maximize the calorific value of coffee grounds and cotton straw after co-torrefaction, Tadesse [19] investigated the effect of reaction temperature, blend composition, and particle size on RSM and found that the optimal operating conditions were 300 °C reaction temperature, 33% coffee ground blending ratio, and 100–125 μm particle size, which yielded a calorific value of 23.955 MJ/kg and mass yield of 40.841%; moreover, the blended biochar had finer texture, better grindability, and better physicochemical and comparative thermal properties.

However, although co-torrefaction can improve the fuel performance of biomass, problems, such as soft texture, inconvenient transportation, and feeding to the furnace are observed; therefore, pressing the biomass into briquettes can improve its applicability. To further improve the practical application potential of torrefied biofuels, studies on torrefied pellets are also being conducted.

Pelletization refers to the compaction of biomass waste by changing its irregular size and structure to solid briquettes, which have high density and combustion potential [20]. Fuad [21] studied the torrefaction of fruit waste and HDPE and found that the calorific value of biochar increased significantly by adding HDPE, which could adhere to the surface of the biomass as a binder after heat liquefaction, thus, improving the adhesion capability of the briquettes. Furthermore, HDPE could significantly increase the density and compressive strength of straw briquettes, with the highest and lowest compressive strengths being 5.3 MPa and 3.5 MPa, respectively, which were higher than those reported in previous studies. Emadi [13] also reported that adding 6% LDPE to the mixed biochar increased the density of wheat and barley by 1.8% and 1.7%, respectively, while adding 10% LDPE to the mixed biochar increased the compressive strength of the wheat and barley briquettes by 280% and 253%, respectively, thus greatly improving their transport and storage efficiency. Portilho [22] evaluated the physicochemical properties of eucalyptus, pine, coffee grounds, and sugarcane bagasse after torrefaction and reported that torrefaction improved biochar fuel properties and pelletization at 10.34 MPa pressure, improved the mechanical properties, and increased the energy potential of the briquettes.

At present, there are few studies on the optimization of co-torrefaction using the response surface method, and the optimization objectives are relatively simple. There are few evaluation indicators of fuel properties that consider the impact of energy/mass yield and ash content at the same time. Furthermore, there is a lack of research on co-torrefaction of FB and PP and on fuel characteristics after the co-torrefaction of PP and lignocellulosic biomass. Considering the potential of FB in energy thermal conversion and the current emerging problem of plastic waste pollution, this study aimed to obtain a clean biofuel with high calorific value and low AC using FB and polypropylene (PP) plastic waste as raw materials and develop an efficient and clean treatment method for plastic waste. The effects of mass/energy yield, high calorific value, and AC were considered simultaneously using a response surface center composite design (CCD). The purpose of using two sets of response factors separately was to study the effect of simultaneously maximizing the MY and HHV or maximizing only the EY. Fluidized torrefaction was conducted in a vertical furnace and the fuel properties and chemical functional group changes of the target product were investigated by ultimate analysis, industrial analysis, and solid-phase infrared analysis. Finally, perspectives on the processing of the acquired clean fuel into briquettes for practical production applications have also been presented. This study will contribute to the sustainable development of biomass resources and the rapid treatment of plastic waste.

## 2. Results and Discussion

### 2.1. Optimization of Biochar Co-Torrefaction

#### 2.1.1. Properties of FB and PP

Table 1 lists the results of the ultimate analysis, HHV, and AC of the dried FB and PP samples. FB had AC, whereas PP had high C and H content but low O content, along with HHV and no AC. Zhang [23] found that torrefaction at low temperatures (200–240 °C) did not significantly increase FB HHV. Oyebode [24] found that torrefaction increases the AC of biochar proportionally with increasing temperature. High AC not only affects the heat generation of the material but also causes slagging and corrosion in boilers during practical applications [25]. Therefore, we combined the biomass waste of FB with PP, having high HHV and low AC, to produce a bioenergy resource for large-scale practical applications.

#### 2.1.2. Statistical Evaluation of the Relationship between Factors and Responses

The CCD in RSM is widely used for analysis, prediction, and optimization in studies involving factors and responses [26]. Based on the experimental results in Table 2, the software provides different fitted models, equations, and statistical values for each response (Table 3). All models showed significant responses (*p* < 0.05). In terms of fitting statistics, the R^2^adj coefficient was more suitable for comparing the adequacy of the models [27] as it indicates a good fit of the regression model. Furthermore, the difference between R^2^pred and R^2^adj was <0.2, implying that the developed model can better reflect the true relationship between factors and responses [26]. Ideally, the coefficient of variation value should be <10%. All the coefficients of variation of the responses met this requirement, indicating that the reproducibility of the experimental results was satisfactory. Moreover, the accuracy of all models met the accuracy requirements of >4 [18], implying a suitable signal-to-noise ratio and indicating that a response regression model can be used to navigate the design space. Table 3 evaluates the reliability of the four parameter models MY, HHV, AC, and EY, and the results prove that the model and equation of each parameter are suitable.

**Figure 1:**
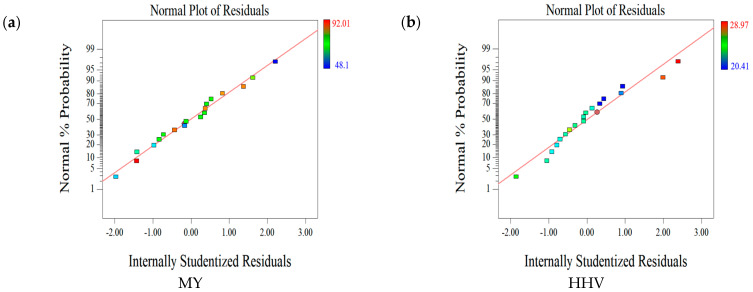

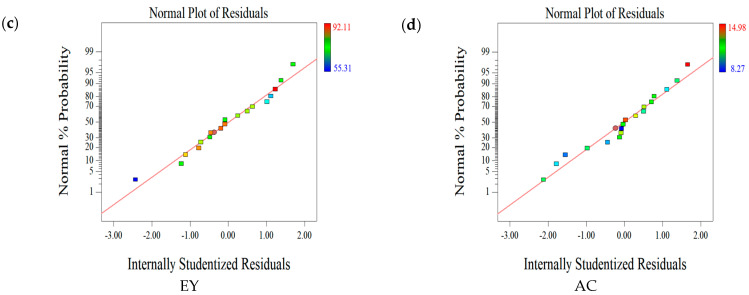
Residual analysis of (**a**) mass yield (MY), (**b**) higher heat value (HHV), (**c**) energy yield (EY), and (**d**) ash content (AC).

Figure 1 shows the residual analysis of HHV, MY, EY, and AC, which can be used to check the reliability of the mathematical model. The experimental values of the vast majority of the four dependent variables were close to the theoretical values, and many points coincided with the straight line, indicating a good agreement between the model and the experimental data. Therefore, bias in the results was negligible.

To predict responses, we used initial unit values for each factor. Table 3 lists the fitting equations of MY, EY, HHV, and AC fitting equations, respectively, where A, B, and C represent the reaction temperature, reaction time, and PP blending ratio, respectively. These equations provide the response values for the co-torrefaction of FB and PP. The positive and negative signals of the equations represent the facilitative and inhibitory effects of the influencing factor on the response, respectively, with + representing the facilitative effect and—representing the inhibitory effect. For MY, both A and B reflected the inhibitory effect, which followed the results of Guo [17]. MY decreased as the reaction temperature and reaction time increased. Furthermore, C represents the promotion effect, which may be related to the physical changes that occur in PP under torrefaction conditions, thus affecting its yield. The regression equation shows that the EY factors had a similar effect on the responses as MY. For HHV, all of A, B, and C reflected the promotion effect, wherein the PP blending ratio had the greatest effect, followed by the reaction temperature, and finally the reaction time. Furthermore, both A and B showed a facilitative effect on the increase in AC, whereas C showed an inhibitory effect, possibly because PP contains almost no ash. According to the regression equation, the effect of B was small for the four responses (HHV, MY, EY, and AC). Valdze [28] reported a similar torrefaction of oat husks. Therefore, the effect of reaction time was neglected in the subsequent response surface analysis, and only the effects of reaction temperature and PP blending ratio were compared and analyzed.

#### 2.1.3. Effect of Parameters on the First Set of Responses (MY-HHV-AC)

**Figure 2:**
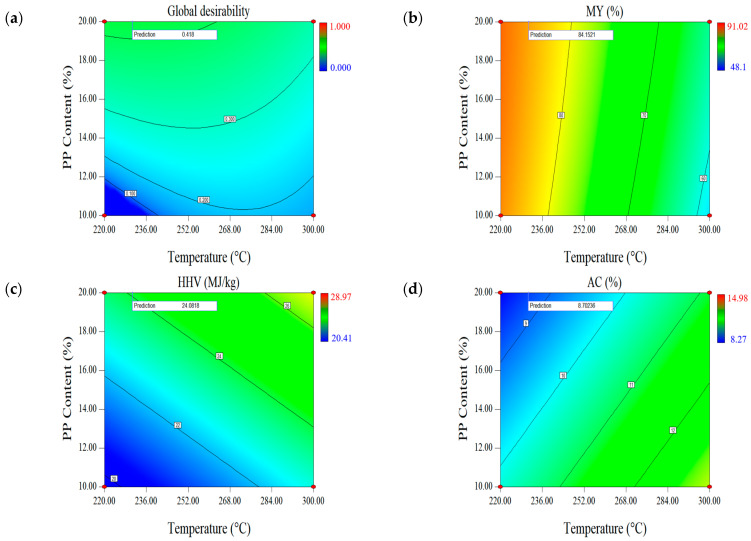
Contour plots for global desirability and the MY-HHV-AC response set: (**a**) Global desirability, (**b**) mass yield (MY), (**c**) higher heat value (HHV), and (**d**) ash content (AC).

The reaction temperature and PP blending ratio showed significant effects in all models (Figure 2). PP is used as a homopolymer, and its melting point is approximately 166.5 °C [29]. As the PP blending ratio increased, the MY of mixed biochar increased, because under low-temperature torrefaction conditions (192.73–327.27 °C), the plastic could not volatilize sufficiently and was affected by temperature. Furthermore, as the reaction temperature continued to rise beyond the PP melting point, the solid PP particles became a sticky liquid polymer coating around the surface of the mixed sample, causing some physical interference with biomass degradation [30]. Therefore, adding PP slowed down the volatilization of cellulose, hemicellulose, and lignin from the lignocellulosic FB biomass, thus reducing the mass loss. Moreover, the experimental results indicated that MY increased proportionally with an increase in the reaction temperature, possibly because as the reaction temperature increases, more solid PP is converted into liquid films, indicating evident liquefaction. Therefore, the MY value of blended biochar was expected to increase further with higher PP blending ratios.

Regarding HHV, the PP blending ratio positively influences the increase in HHV and is the most important parameter affecting the changes in HHV. The higher the PP blending ratio, the higher the heat value of the mixed biochar, and this trend increased significantly as the PP blending ratio increased. This was mainly because the raw material had HHV ~2.5 times higher than FB. Kim [31] also obtained similar calorific value results while studying the properties of low-temperature pyrolysis on PP and polystyrene plastics. Therefore, when the PP blend ratio in the mixture was high, the produced mixed biochar showed a large HHV.

The PP blending ratio also positively influenced AC, possibly because of the AC of the raw materials. The industrial analysis results (Table 2) showed that PP was almost ash-free. Previous studies found that as the reaction temperature increases, the AC of lignocellulosic biomass increases to varying degrees [32,33], and high AC causes problems such as corrosion and slagging of equipment. Therefore, torrefaction of a mixture of ash-containing FB lignocellulosic biomass with organic polymer materials, with less AC, is a reliable method for producing biofuels with low AC.

We expected the biomass of FB to volatilize before that of PP in this study. Moreover, the volatilization and decomposition of PP are very small [30]; therefore, the hemicellulose, cellulose, and lignin in FB undergo changes mainly during the co-torrefaction process. Furthermore, the reaction temperature, which supplies energy, is the most important factor in torrefaction [34]. Here, as the reaction temperature increases, the rate of mass loss of the mixed biochar increases, which increases proportionally with the increase in the reaction temperature (Figure 2). During torrefaction, the mass loss of the sample is mainly divided into three stages. (1) When the reaction temperature is <200 °C, the samples are dehydrated, and a small quantity of light volatiles are removed, and at 200 °C, hemicellulose degrades, mainly by deacetylation and depolymerization [35,36]. At this temperature, amorphous cellulose also degrades with partial demethoxylation of lignin (eugenol base) [37]. (2) At ~250 °C, large-scale cellulose degradation begins, crystallized cellulose degrades, and lignin depolymerizes. (3) At 300 °C, cellulose degrades, lignin fat side chains break, and most hemicellulose is degraded [38]. Elder and Soltes [39] also found similar results; specifically, they reported that hemicellulose is extremely sensitive to temperature and decomposes in the temperature range of 200–260 °C, and lignin is decomposed only at a temperature range of 280–500 °C. Therefore, at higher reaction temperatures, the mass loss of the mixture will be greater, which is mainly because of the large-scale decomposition of lignin in the biomass. In this study, the reaction temperature was the second most important factor affecting HHV. Furthermore, the effect of torrefaction on HHV may be related to the degradation of biomass—lignin showed the largest HHV for FB [40]. However, as lignin is more stable than cellulose and hemicellulose, an increase in reaction temperature will mainly degrade hemicellulose and cellulose, whereas lignin will mostly be retained [41]. PP does not decompose at this temperature, and most organic macromolecules are retained. Therefore, as the reaction temperature increases, HHV also increases at different degrees. This could also be explained from another perspective: an increase in reaction temperature promotes decarboxylation and dehydration, thus increasing the carbon content of biochar, in turn increasing HHV [42]. Furthermore, the AC of the mixture increased with increasing reaction temperature, mainly because the percentage of fixed carbon and AC increased to some extent as the volatiles in the FB and PP continued to decrease as torrefaction proceeded (the volatile and fixed carbon of the MY-HHV-AC set accounted for 74.12% and 17.35%, respectively, while the volatile and fixed carbon of the EY-AC set accounted for 74.96% and 16.82%, respectively). Therefore, we determined the optimal reaction conditions that minimize AC to mitigate or avoid adverse phenomena such as slagging and corrosion of boiler equipment during the combustion of mixed biochar.

The contour graphs for global desirability and MY-HHV-AC response are given in Figure 2. The highest MY was obtained at the lowest reaction temperature and the highest PP blending ratio, whereas the largest HHV was obtained at the highest reaction temperature and the highest PP blending ratio. Last, the lowest AC was obtained at the lowest reaction temperature and the highest PP blending ratio. Multiple combination scenarios were created based on the operating conditions and corresponding predicted responses, and depending on the desired optimization, the software assigned a global expectation value to each set of MY-HHV-AC. The results showed a maximum global expectation value of 0.418 for all combinations, whose predicted responses of MY, HHV, and AC were also marked in the corresponding contour plots. Surface responses were also generated for the global expectation function to visualize all possible combinations in the design space. The corresponding contours are reflected in Figure 2. The operating conditions, simulated responses, and actual responses at the maximum global expectation are shown in Table 4.

Considering the insignificant effect of the reaction time and the negative effect of the longer reaction time on MY and AC, the shortest reaction time was selected, which could reduce the energy consumption of the equipment and ensure economic experimentation. For the first set of responses (MY-HHV-AC), the optimal operating conditions were at a reaction temperature of 230.68 °C, a reaction time of 30 min, and a PP blending ratio of 20%. The validation results succeeded for the predicted values because they were within the acceptable error of ±10% of the predicted responses (0.15%, 0.21%, and 1.95% for MY, HHV, and AC, respectively) [42].

#### 2.1.4. Effect of Parameters on the Second Set of Responses (EY-AC)

Considering that MY and HV were included in the set of calculations of EY, the EY-AC response was investigated to determine the effect of using EY instead of MY and HHV on the optimal factors. As maximum energy efficiency was required after torrefaction, the EY response was proposed to be maximized, while AC could be minimized.

According to a previous study, the significance of the effect of reaction temperature and PP blending ratio on each response was different, with the effect of reaction temperature and PP blending ratio significant for MY and HHV, respectively. At higher reaction temperatures, MY was low but HHV was improved, whereas at lower reaction temperatures, MY was high but HHV was relatively low. In contrast, the trend of EY was always similar to MY at both low temperature (220 °C) and high temperature (300 °C), and EY was comparatively more strongly influenced by MY. These results were consistent with the results of previous studies [43].

The effect of the PP blending ratio and reaction temperature on the changes in EY differed. An increase in the PP blending ratio benefited both MY and HHV. When the reaction temperature was 220 °C and the PP blending ratio increased from 10% to 20%, EY increased from 87.88% to 88.73%, which was less than 1%; contrastingly, when the reaction temperature was 300°C and the PP blending ratio increased from 10% to 20%, EY increased from 64.23% to 76.16%, which was 11.93%. These results indicated that the effect of the PP blending ratio on the EY was significantly lower than the effect of the reaction temperature; additionally, the changes in the EY gradually stabilized as the reaction temperature decreased. Because the changes in the PP blending ratio had the most significant effect on HHV, this conclusion can also explain how the effect of MY on EY was greater than the effect of HHV.

**Figure 3:**
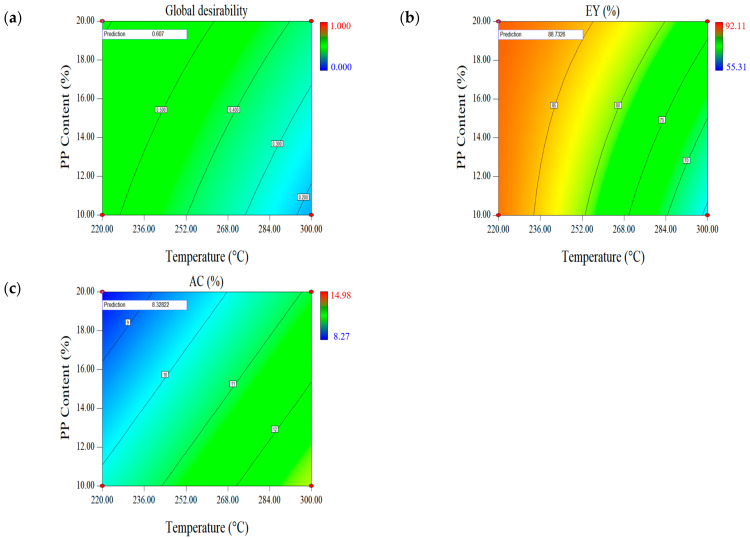
Contour plots for global desirability and response set EY-AC: (**a**) Global desirability, (**b**) energy yield (EY), and (**c**) ash content (AC).

Figure 3 shows the curve contours of the simultaneous optimization of EY and AC. Among the optimization results, the maximum value of global desirability was 0.607, corresponding to the optimal operating conditions of 220 °C reaction temperature, 30 min reaction time, and 20% PP blending ratio. The results of the predicted and experimental values are listed in Table 5. Compared with the predicted values, the errors of EY and AC were 0.63% and 1.32%, respectively, indicating satisfactory simulation results.

Furthermore, we compared the HHV, EY, and AC of the mixed biochars torrefied under optimal operating conditions with common torrefied biochars (rice husk, peanut husk, wheat straw, and bamboo residue) (Table 6). The optimized mixed biochars had high EY (up to 92.9% in the MY-HHV-AC set) and almost the highest HHV with the lowest AC. Therefore, the results of the study demonstrated that the mixed biochars obtained by torrefaction after optimization with RSM had considerable potential as a renewable energy source.

These results and discussion indicated that RSM was successfully used to perform multiobjective optimization of the co-torrefaction process of FB and PP; additionally, high-quality biofuels were obtained under the optimal operating conditions corresponding to different response combinations. In Section 2.2.1, the biofuel properties of the two sets of optimal mixed biochars have been characterized.

### 2.2. Characterization of the Optimal Co-Torrefaction Biochar

#### 2.2.1. Fuel Characteristics

**Figure 4:**
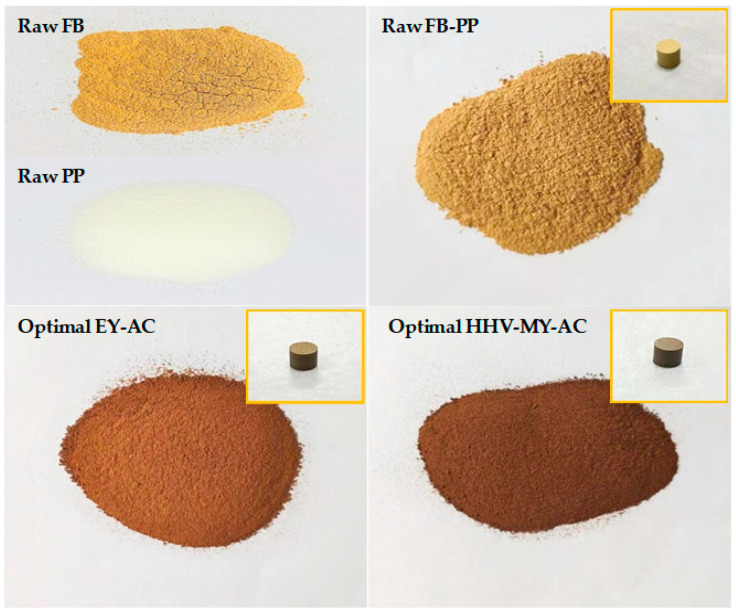
Images of raw fungal bran (FB), raw polypropylene (PP), raw FB-PP, optimal EY-AC, and optimal HHV-MY-AC.

Figure 4 shows the mixed biochars prepared under two sets of optimal operating conditions and the original samples. The surface of PP powder was smoother than pure FB powder, and the color of the mixed sample, with scattered white PP particles after thorough mixing, was like that of pure FB. On comparison of the optimized mixed biochars under different operating conditions, the color of the EY-AC set was slightly lighter than the MY-HHV-AC set, possibly because the latter was torrefied at a higher temperature and charred more deeply. Furthermore, compared with the blended original samples, the two optimized sets having a finer texture and better grindability could be easily converted into molded particles without adding humectants. The outer surface of the particles in the optimized set was smoother and more uniform than that of compressed molded particles.

**Figure 5:**
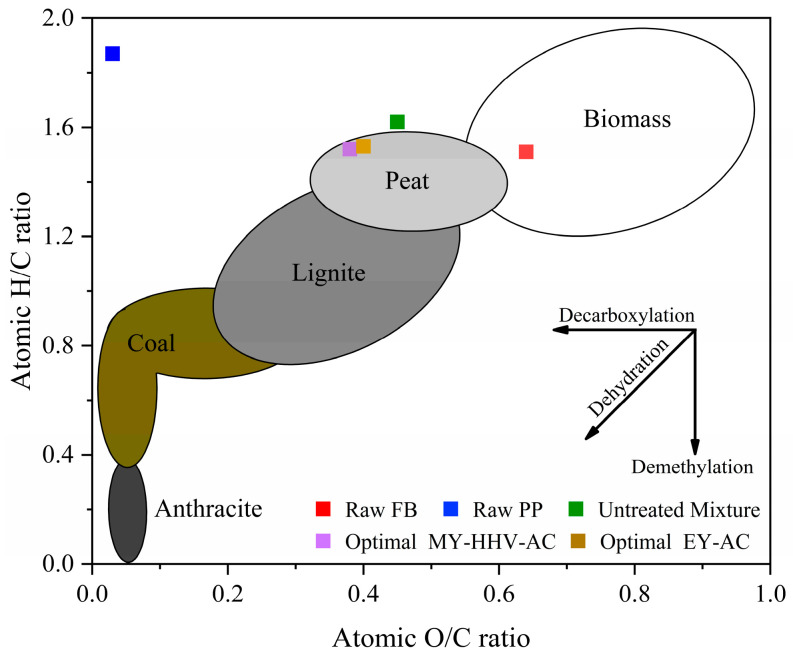
Van Krevelen diagram of H/C and O/C atomic ratios of the raw and optimal samples.

Figure 5 shows the analysis results of the H/C and O/C atomic ratios of the untreated mixed sample and two sets of optimized samples and their comparisons with other solid fuels using a van Krevelen diagram, which is a method to interpret the properties of solid fuels based on elemental content. In the diagram, solid fuels with better fuel performance were located near the origin [48]. Furthermore, the H/C and O/C ratios of the two sets of samples after optimized torrefaction were lower than the untreated mixed sample, because dehydration, decarboxylation, and deoxidation mainly occurred during torrefaction [49], thus indicating that torrefaction can effectively reduce the H/C and O/C ratios of the mixed samples. Huang [50] also reached the same conclusion while studying food waste torrefaction, where although the H/C (1.52) and O/C (0.38) ratios of the MY-HHV-AC set were lower than those of the EY-AC set (1.53 and 0.4, respectively), the difference was not significant, possibly due to the small difference in the torrefaction temperature between the two sets. In this study, the properties of the two optimized samples were similar to those of peat and lignite, and at higher torrefaction temperatures, the properties would be similar to those of lignite and bituminous coal. These results showed that the biochar prepared by combining FB and PP can serve as a suitable fuel.

**Figure 6:**
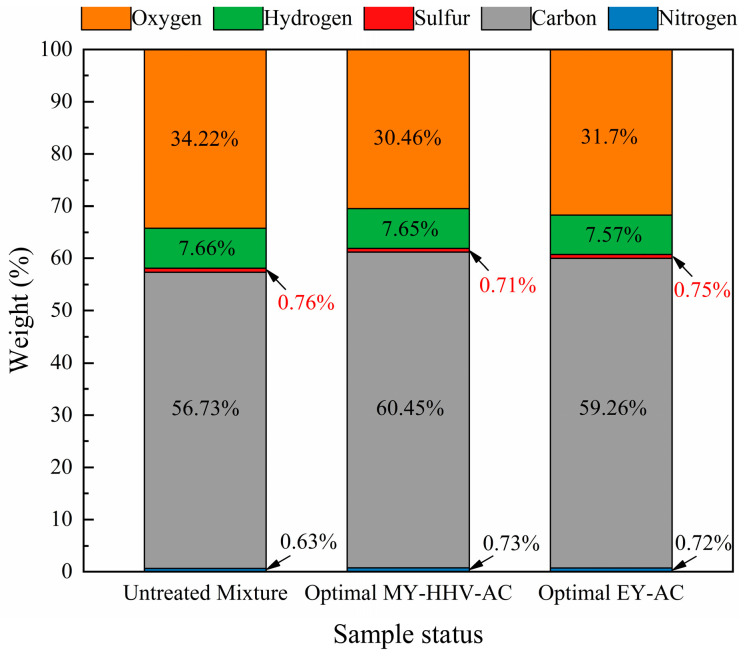
Compositions of raw and optimal samples.

The results of the elemental analysis of the untreated mixed raw sample and the two optimized sets of samples are shown in Figure 6. The optimized MY-HHV-AC set had the highest C content (60.45%) and the lowest O content (30.46%), whereas the optimized EY-AC set had a higher C content (59.26%) than the original blended mixture (56.73%) but a lower C content than the MY-HHV-AC set; additionally, the O content (31.7%) was lower than the blended original sample (34.22%) but higher than the MY-HHV-AC set, which is attributed to the thermal degradation of the volatile components, such as acids, alcohols, aldehydes, and oxygenated ketones, in the mixed biochar as the temperature increased [51]. In contrast, the H content remained stable, due to the low intensity of torrefaction (the optimized torrefaction temperatures of the two sets were 230.68 °C and 220 °C, respectively); similarly, the N content remained stable. However, the S content decreased with increasing torrefaction temperature, possibly due to the large amount of Ca in FB. FB biomass contains high Ca amounts. However, to remove miscellaneous bacteria while preparing the FB culture matrix, quicklime, whose major component is CaO, is added. While studying the cocombustion of plastics with printing and dyeing sludge, Ding [52] found that CaCO_3_ in the plastic additive, as a calcium-based sulfur-fixing agent, chemically reacts with the sludge to generate CaSO_4_; moreover, more S is fixed in the bottom slag to realize the self-desulfurization effect. The related chemical reactions are given by Equations (1) and (2).

(1) $${\mathrm{CaCO}}_{3}\to {\mathrm{CaO}+\mathrm{CO}}_{2}$$

(2) $${2\mathrm{CaO}+2\mathrm{SO}}_{2}{+O}_{2}\to {2\mathrm{CaSO}}_{4}$$

Therefore, we speculated that the reduction of the S content may be related to the large proportion of Ca in the FB biomass. Thus, owing to the practical importance of the combustion of biochar through the co-torrefaction of FB and PP, the combustion characteristics and gas pollutant emissions of pelleted biochar will be studied in the future.

**Figure 7:**
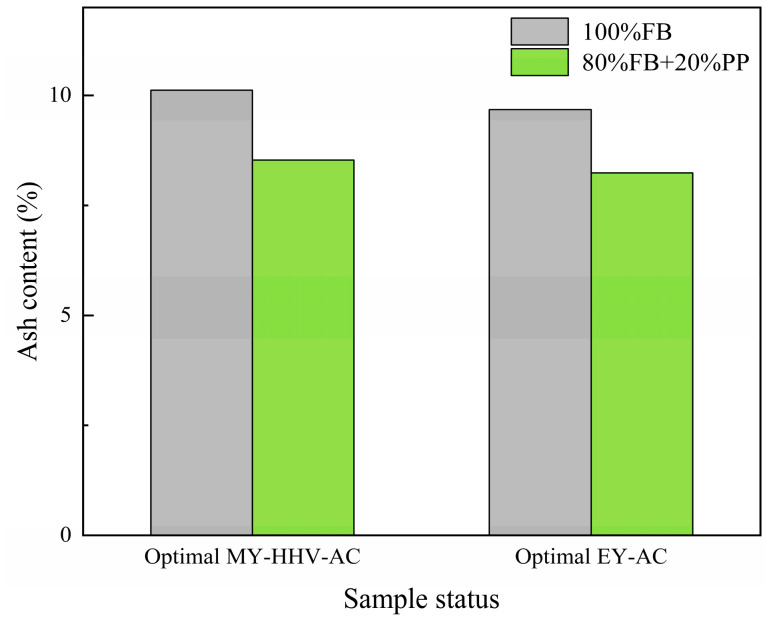
Ash content of 100% FB and mixed 20% PP under two sets of optimized conditions.

To compare the effect of PP additives on the AC of biochar, we measured and compared the AC of pure FB biochar torrefied under two optimized conditions (Figure 7). Similarly to a previous study, AC increased with increasing torrefaction temperature. Under the optimized conditions of the MY-HHV-AC set, the AC of the pure FB biochar was 10.12%, and when 20% PP was blended, the AC of the mixed biochar decreased from 15.71% to 8.53%. Furthermore, under the optimized conditions of the EY-AC set, the AC of the pure FB biochar was 9.68%, and when 20% PP was blended, the AC of the mixed biochar was reduced by 14.88% to 8.24%. Thus, after blending PP, the AC reduction rate of the two sets differed by <1%, which could be attributed to the variable temperature difference of <11 °C. These results confirmed that blending PP additives can effectively reduce the AC in the final product, which can reduce slagging and corrosion in the boiler reactor and improve the combustion efficiency.

#### 2.2.2. Fourier Transform Infrared (FTIR) Analysis

**Figure 8:**
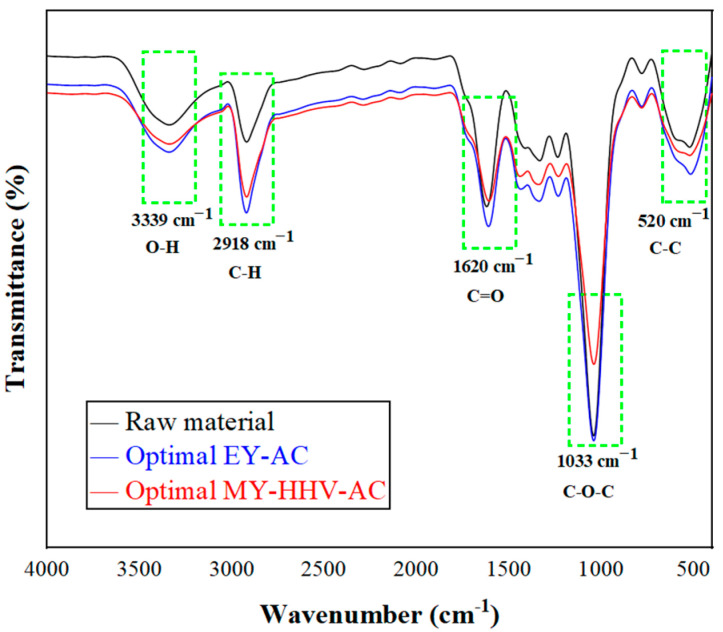
Fourier transform infrared spectra of raw material and the response sets after torrefaction.

To study the effect of torrefaction on the chemical structure of the lignocellulosic biomass and the organic polymer material blends, the chemical changes of the functional groups after torrefaction were investigated using attenuated total reflectance (ATR)-FTIR for the mixed feedstock of FB-PP and two sets of optimal working conditions, as shown in Figure 8.

The broad peak in the absorption range of 3339 cm^−1^ indicated the stretching vibration of O-H through intra- and intermolecular hydrogen bonds [53], mainly in phenolic and aliphatic structures, where O-H stretches to the hydroxyl group and dehydroxylation and condensation decrease the corresponding peak [54]. As PP was dried and dehydrated before experimentation, the PP water molecular vibration had no effect, which was confirmed by comparing the FTIR spectra of pure PP and the experimental PP. The peak at 2918 cm^−1^ was attributed to the C-H stretching vibrations of alkanes in hemicellulose and cellulose [55] and the stretching vibrations of olefinic and aromatic C-H groups in PP; moreover, the intensity of this peak decreased with increasing torrefaction intensity, which was like previous findings [56]. The intensity of the peak at 1620 cm^−1^, which was mainly due to the tensile vibration of carbonyl bonds (C=O) in cellulose and hemicellulose [57], decreased with an increasing intensity of torrefaction. Furthermore, the peak at 1033 cm^−1^ was mainly due to the C-O-C stretching vibration of cellulose and hemicellulose [58], and the lowest transmittance of MY-HHV-AC was observed with higher reaction temperature in this range, indicating that at higher temperatures, low cellulose and hemicellulose fractions suffered the most. This was consistent with the results reported by Gan [26], who stated that the depletion of hemicellulose formed more nonpolar and unsaturated compounds in the carbonized samples. The reduction of hemicellulose and the enrichment of lignin increased the uniformity of carbonized samples [59]. Furthermore, the peak value changes were most significant at the 1033 cm^−1^ band, because the characteristics of polysaccharides (mainly cellulose and hemicellulose) led to the most changes in the 1200–800 cm^−1^ region [60], and as the reaction temperature increases, the peak intensity decreased because of dehydration decarboxylation of hemicellulose during deacetylation of carbohydrate consumption and ester group removal [61]. In the 1400–1190 cm^−1^ region, vibrations, mainly C=C aromatic ring vibrations, stretching vibrations of acyl aromatic C-O bond [61], shear vibrations of saturated alkane-CH2-, and deformation vibrations of -CH3 related to lignin and saturated PP alkanes were also found [62]. Insignificant peak fluctuation indicated that the decomposition of lignin and saturated alkane of PP was not obvious under all temperature conditions and the peak at 520 cm^−1^ was caused by the stretching of the aromatic ring C-C [63]. In addition, oxygenated compounds gradually decomposed during the torrefaction process, and cellulose and hemicellulose decomposed at different degrees. The mixed biochar had low organic matter content and O content, and although different degrees of quality loss were observed, the mixed biochar hydrophobicity was enhanced. Moreover, the mixed biochar under the optimal operating conditions of the two sets maintained the performance of the feedstock, with the MY-HHV-AC set showing maximum cellulose and hemicellulose decomposition, the highest lignin enrichment, and the largest HHV, thus being consistent with the previously discussed results.

Through the comparison of the response surface multiobjective optimization analysis results and the fuel characteristics of the optimization set, it was found that the ash content of the MY-HHV-AC set was only 0.31% higher than that of the EY-AC set, while the energy yield of the MY-HHV-AC set was significantly increased to 92.9% from 88.17%. At the same time, the samples of the MY-HHV-AC set have better fuel characteristics; therefore, we believe that the biochar effect of the MY-HHV-AC optimization set is superior.

## 3. Applications and Discussion

### 3.1. Densification of Co-Torrefaction Biochar

The results discussed in the previous sections showed that the research method proposed in this study could successfully prepare high-quality biofuels; however, some disadvantages were observed. For example, biomass fuel was soft and difficult to transport, and transport materials had a short shelf life. These problems can be overcome if biomass fuel is processed into briquettes. Furthermore, according to previous research, plastic can act as a binder during the blending process with biomass; therefore, because briquettes can show better mechanical properties, we studied the bonding and pelleting process of FB and PP under torrefaction conditions.

**Figure 9:**
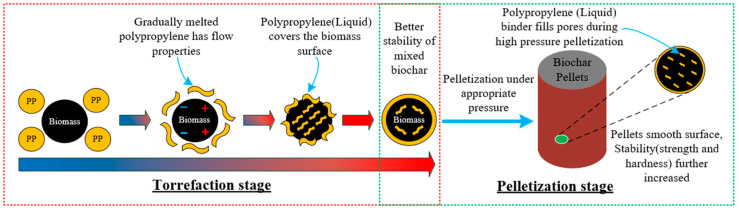
Principle of biochar pelletization using PP as a binder at different stages.

Figure 9 shows the pelletization mechanism of PP as a binder and FB biomass. During torrefaction, as the reaction temperature increases, PP is easily transformed from solid particles to a liquid film with a certain viscosity. The liquid gradually surrounds the biochar surface and continuously fills the size pores of the biomass matrix. Consequently, increased contact surface area further enhances the liquid bridge and attractive forces between adjacent particles [64]. The FTIR results showed that the presence of oxygen-containing groups on the surface of mixed biochar also promoted strong electrostatic attraction, including H bonding and van der Waal’s forces [65]. Furthermore, the interaction of cohesive and viscous forces between particles bound the biochar and PP particles tightly and stably. Moreover, to further realize practical production applications, we prepared briquettes of the mixed biochar into briquettes through pressurization. This pelletization causes mutual diffusion in the raw material, which consequently results in the formation of strong solid bridges [65]. In addition, liquid PP has certain fluidity under high pelletization pressure. Pressurization allows the liquid PP to penetrate the porous space better inside the biochar particles, thus establishing a strong bond [66]. These chemical interactions between biochar and PP establish a good chemical bridge and improve the binding capacity and stability of the particle matrix. Corresponding tests will be conducted to determine the moisture absorption, compressive strength, hardness, and density of the briquettes, and the bonding mechanism during pelletization.

### 3.2. Biofuel Recycling Economy

**Figure 10:**
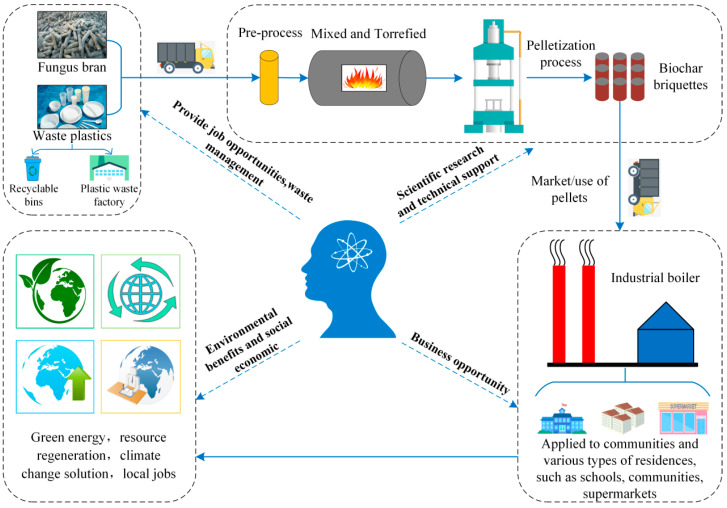
Biofuel circular economy framework.

In this study, we proposed a method to prepare a novel fuel, having HHV and low AC, through the torrefaction of FB and PP waste plastic. The proposed method provides novel information for the development, heat conversion, and utilization of renewable energy from plastic waste, and the resulting biomass mixture can serve as an alternative to fossil fuels. With the backdrop of sustainable development, the circular economy has been gaining increasing attention. The circular economy considers all types of domestic and industrial waste as secondary raw materials and realizes the regeneration of resources. Inspired by the study of Sharma [67], the structural framework of the biofuel cycle economy is presented in Figure 10. FB is recycled in farms, whereas PP waste plastic is recycled through plastic scrap plants and recyclable bins along with other bulk waste. After recycling, the waste is transported to a biomass waste heat treatment plant, where it is crushed, mixed, torrefied, and pelletized to produce high-performance solid clean fuel. The manufactured clean solid fuel is then sold to major industries for use in boilers to replace conventional fossil fuels; in addition, it is used as an energy source in schools, communities, and supermarkets. This solid fuel mainly comprises a large amount of waste biomass and a little waste plastic; thus, it is appropriately categorized as green energy. In addition, manufactured fuel provides an efficient disposal strategy for waste that would otherwise be landfill or incinerated, thus effectively preventing the release of toxic and harmful gases, reducing the damage to human health and the environment, and mitigating global warming. Finally, the bioenergy supply chain can also provide more work opportunities. In addition, to increase the economic viability of the bioenergy supply chain, additional revenue can be generated by charging fees from waste generators [66]. Although a technique to manufacture high-performance solid clean fuel, which has several environmental and economic benefits, was proposed in this study, some limitations still exist. Thus, future research should conduct comprehensive technical and economic analysis and life cycle analysis and expand the scope of studies to the selection of materials that can increase the efficiency of the biofuel circular economy structure.

## 4. Materials and Methods

### 4.1. Materials

FB samples were recovered from Huang Songdian fungus plantations in Jilin Province, China. As the fungus is cultivated in an aquatic medium, FB developed immediately after ear emergence has a high water content and is thus predried before torrefaction pretreatment. After removing the FB from the plastic bag, it was air-dried, cut into small pieces of 3–5 cm (length), oven-dried at 105 °C for 24 h, and finally crushed into particle sizes of 0–0.15 mm. Discarded plastic spoons and lunch boxes collected from a nearby restaurant were washed and then used as PP waste plastics. These waste plastics were sliced to 0.5 cm and then crushed into a powder with a particle size of 0–0.15 mm using a grinder. Subsequently, both dehydrated FB powder and waste plastic powder were sealed in separate plastic bags and stored in a desiccator at 25 °C until further analysis.

### 4.2. Torrefaction Experiment

**Figure 11:**
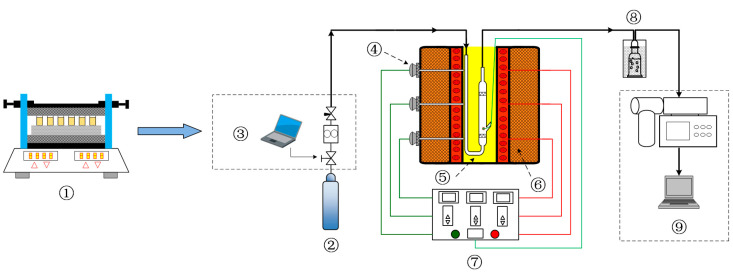
Schematic diagram of the experimental system: ① mechanical oscillator, ② Ar, ③ mass flow controller, ④ thermocouple, ⑤ reactor, ⑥ furnace, ⑦ temperature controller, ⑧ iced acetone bath, ⑨ mass spectrometer.

Our preliminary experiments demonstrated that owing to the high viscosity of PP after being melted using heat, it was not easy to collect samples because of the action of the viscous polymer, and it was easy to damage the sieve plate of the reactor. Therefore, we designed a fluidized torrefaction reactor for the torrefaction experiment (Figure 11). Mixed samples (5 ± 0.001 g) were weighed in proportion for each experiment, stirred mechanically using an oscillator for 20 min to ensure homogeneous mixing, and then placed in the fluidization torrefaction reactor. Argon gas was used in the reactor at a pressure of 0.3 MPa and the flow rate was set at 2.5 L/min, as determined through a cold fluidization experiment. An electric heating furnace was used to heat the reactor from room temperature to the specified reaction temperature at a heating rate of 10 °C/min. A thermocouple was used to measure the temperature, and the central temperature of the reactor was recorded using a digital temperature sensor, with an accuracy of 0.1 °C, at a time interval of 1 s. Ice acetone was used to absorb condensable volatiles at the reactor outlet and obtain tar, and MS was used to monitor noncondensable volatiles online. Liquid and gas phase products will be discussed in future work. After the reaction was complete, the reactor was removed from above the furnace and cooled to room temperature. Then, the sample was weighed accurately to 1 mg. Dry samples were placed in PE sealed bags for further testing.

### 4.3. Optimization of the Torrefaction Process

The RSM of CCD was used to optimize the torrefaction process using reaction temperature, reaction time, and PP blending ratio for two sets of response factors: (1) mass yield (MY)-higher heating value (HHV)-AC and (2) energy yield (EY)-AC. Often, 0.5–20 wt% or more of binder is added to the biomass [68]; therefore, a 10–20% PP blending ratio was selected in this study. As CCD was rotatable at the outer edge, the ranges of reaction temperature, reaction time, and PP blending ratio were 192.73–327.27 °C, 19.77–70.23 min, and 6.59–23.41%, respectively (Table 7).

In the first set of reactions, both MY and HHV were maximized, whereas AC selection was minimized; in the second set of reactions, EY was maximized, and AC was minimized. Furthermore, MY and EY were calculated according to Equations (3) and (4), respectively [69]. Based on the Chinese standard GB/T213-2008, HHV was determined using an SDC311 oxygen bomb calorimeter (Suntech, Changsha, China), and each group of samples was analyzed twice to obtain an average value to increase the accuracy of the experimental results.

(3) $$\mathrm{Mass}\;\mathrm{Yield}\left(\%\right)=\mathrm{Weight}\;\mathrm{of}\;\mathrm{torrified}\;\mathrm{biomass}/\mathrm{Weight}\;\mathrm{of}\;\mathrm{raw}\;\mathrm{biomass}\times 100\%$$

(4) $$\begin{array}{l} \mathrm{Energy}\;\mathrm{Yield}\left(\%\right)=\mathrm{Mass}\;\mathrm{Yield}\times \\ (\mathrm{HHV}\;\mathrm{of}\;\mathrm{torrified}\;\mathrm{biomass}/\mathrm{HHV}\;\mathrm{of}\;\mathrm{raw}\;\mathrm{biomass})\times 100\% \end{array}$$

To develop a model expressing the relationship between the above factors and the response, Design Expert V8.0.6.1 software was used to evaluate model performance by analysis of variance (at 95% confidence level), fit statistics, and diagnostic plots. If responses are analyzed individually, different optimal conditions can be determined [70]. In this study, multiple objectives were optimized simultaneously to obtain an optimal operating condition for each set of responses and the optimal operating conditions were experimentally validated to assess the reliability of the model.

### 4.4. Characterization of the Optimal Co-Torrefaction of Biochar

The percentages of the mass of ash, volatile fraction, and fixed carbon in the samples were obtained based on the Chinese standard GB/T30732-2014, measured using an SDLA718 industrial analyzer (Sandy, Changsha, China). Furthermore, the mass percentages of C, H, and N in the samples were measured using an EA3000 automatic elemental analyzer (Euro Vector, Italy) based on the Chinese standard GB/T30733-2014. Meanwhile, the mass percentage of S was determined using a SDS350 infrared sulfur analyzer (Sandy) based on the Chinese standard GB/T25214-2010, and the O content was obtained using the differential subtraction method. Solid-phase infrared analysis was performed using a spectrum two portable Fourier transform infrared (FTIR) spectrometer (Pekin Elmer, USA), with a wavelength accuracy of 0.01 cm^−1^.

## 5. Conclusions

In this study, RSM was used to optimize the co-torrefaction of FB and PP waste plastic. The multiobjective optimization of the two sets of responses, MY-HHV-AC and EY-AC, was used to obtain clean fuel solids having HHV and low AC. The main conclusions are as follows:

(1) Under the optimal operating conditions, the HHV of MY-HHV-AC mixed biochar was 24.13 MJ/kg, MY was 84.28%, and AC was 8.53%. Furthermore, in the EY-AC set, the EY of mixed biochar was 88.17% and AC 8.22%. The effect of the PP blending ratio on HHV was greater than the reaction temperature, whereas the effect of the reaction temperature on MY and AC was greater than the PP blending ratio. In addition, EY was more influenced by MY.

(2) The MY-HHV-AC set had the lowest H/C (1.52) and O/C (0.38) ratios. As the reaction temperature increased, the C content of the mixed biochar increased and the O content decreased; in addition, the S content decreased from 0.76 to 0.71, possibly due to the higher Ca content of FB. Furthermore, when 20% PP was added, the AC of the two sets of mixed biochar decreased by 15.71% and 14.88%, respectively.

(3) PP is a promising binder for biofuels. The practical application of solid biofuels can be further improved by torrefaction and then pelletization. Finally, a framework for a biofuel circular economy was established.

Based on practical applications, this research successfully produced clean solid biofuels, having HHV and low AC, to replace conventional fossil fuels, promote the development of renewable energy, and provide research protocols for the pretreatment of biomass and plastic waste for torrefaction.

## Figures and Tables

**Table 1:** FB and PP properties.

Sample	Ultimate Analysis (wt.%, daf.)					HHV	V	FC	AC
	C	H	O ^a^	N	S	(MJ/kg)	(wt.%, db.)	(wt.%, db.)	(wt.%, db.)
FB	49.81	6.25	42.41	0.79	0.74	17.32	72.81	19.7	7.49
PP	82.88	12.9	3.33	-	0.89	44.11	100	-	-

Note: ^a^ By difference: O = 100−C−H−N−S; db. = dry basis; daf. = dry and ash-free basis.

**Table 2:** Design matrix and responses for biochar optimization.

Std. Order	Run Order	Temperature (°C)	Time (min)	PP (%)	HHV (MJ/kg)	MY (%)	EY (%)	AC (wt.%, db.)
1	8	220	30	10	20.49	85.28	87.4	9.05
2	12	300	30	10	22.9	57.02	65.3	12.94
3	11	220	60	10	20.51	84.56	86.71	10.9
4	9	300	60	10	23.13	54.06	62.53	14.45
5	15	220	30	20	23.24	86.38	88.53	8.27
6	6	300	30	20	28.26	62.54	77.95	9.8
7	7	220	60	20	23.26	85.52	87.77	9.42
8	18	300	60	20	28.97	58.1	74.23	10.97
9	2	192.73	45	15	21.59	91.02	92.11	9.86
10	3	327.27	45	15	24.53	48.1	55.31	14.98
11	14	260	19.77	15	22.48	76.85	80.98	10.56
12	17	260	70.23	15	23.53	70.82	78.1	12.96
13	13	260	45	6.59	20.41	70.44	75.34	13.18
14	10	260	45	23.41	26.37	75.58	84.47	10.84
15	1	260	45	15	22.81	72.95	77.99	11.27
16	4	260	45	15	23.36	73.75	80.74	11.98
17	5	260	45	15	22.62	73.39	80.35	11.35
18	16	260	45	15	23.41	73.79	80.96	12.04

Note: HHV = higher heating value; MY = mass yield; EY = energy yield; AC = ash content.

**Table 3:** Fitted model and equations generated by RSM software and statistics for each response.

Parameter	Model	Equation	*p*-Value	Adjusted R^2^	Predicted R^2^	C.V.%	Adequate Precision
HHV	Linear	23.44 + 1.52 × A + 0.2 × B + 1.96 × C	<0.0001	0.852	0.779	3.9	17.043
MY	Quadratic	73.49 − 13.34 × A − 1.4 × B + 1.48 × C − 0.73 × AB + 0.94 × AC − 0.2 × BC − 1.46 × A^2^ + 0.049 × B^2^ − 0.24 × C^2^	<0.0001	0.995	0.983	1.19	70.149
EY	Quadratic	79.95 − 9.69 × A − 0.94 × B + 3.07 × C − 0.63 × AB + 2.77 × AC − 0.13 × BC − 1.96 × A^2^ + 0.099 × B^2^ + 0.23 × C^2^	<0.0001	0.964	0.891	2.33	23.815
AC	Linear	11.38 + 1.4 × A + 0.71 × B − 0.94 × C	<0.0001	0.769	0.654	7.7	14.779

Note: A = reaction temperature; B = reaction time; C = PP blending ratio; AB, AC, and BC represent interaction terms; A^2^, B^2^, and C^2^ represent quadratic terms.

**Table 4:** Validation results for the optimized set of responses of MY-HHV-AC.

Optimal Factors	Units	Data	Responses	MY (%)	HHV (MJ/kg)	AC (wt.%, db.)
Temperature	°C	230.68	Prediction	84.15	24.08	8.70
PP	%	20				
Time	min	30	Validation	84.28 (0.13)	24.13 (0.09)	8.53 (0.02)

**Table 5:** Validation results for the optimized set of responses of EY-AC.

Optimal Factors	Units	Data	Responses	EY (%)	AC (wt.%, db.)
Temperature	°C	220	Prediction	88.73	8.33
PP	%	20			
Time	min	30	Validation	88.17 (0.01)	8.22 (0.04)

**Table 6:** Comparison of various types of biomass waste.

Biomass	Torrefaction Parameter	HHV (MJ/kg)	Energy Yield (%)	Ash Content (db%)	Reference
Rice husk	Raw 220–300 °C, 30–60 min	12.27 13.48–17.88	- 64.82–78.96	15.98 19.71–78.19	Zhang [44]
Corn straw	Raw 275–375 °C, 60–120 min	16.8 16.90–19.80	- 35.4–66.2	6.49 8.36–12.87	Liu [45]
Wheat straw	Raw 250–300 °C, 30 min	17.29 19.17–24.32	- 64.91–80.8	8.14 11.96–13.09	Bai [46]
Bamboo residues	Raw 200–300 °C, 60 min	16.79 17.57–21.96	- 64.72–90.25	12.4 14.98–27.03	Hu [47]
FB-PP	Raw MY-HHV-AC EY-AC	21.89 24.13 23.18	- 92.9 88.17	6.08 8.53 8.22	This study

**Table 7:** Factor levels for the co-torrefaction of fungal bran (FB) and polypropylene (PP).

Range and Levels						
Factor	Units	−α	−1	0	1	+α
Temperature	°C	192.73	220	260	300	327.27
Time	Min	19.77	30	45	60	70.23
PP	%	6.59	10	15	20	23.41

Note: Noninteger temperatures and times are rounded to whole numbers; α is the axis point.

## Data Availability

Not applicable.

## Sample Availability

Samples of the compounds are available from the authors.

## Nomenclature

FB	Fungus bran	HHV	Higher heating value
PP	Polypropylene	MY	Mass yield
AC	Ash content	EY	Energy yield
LDPE	Low-density polyethylene	A	Reaction temperature
HDPE	High-density polyethylene	B	Reaction time
RSM	Response surface methodology	C	PP blending ratio
CCD	Center composite design	α	Axis point
